# Immunological Perspectives of Leishmaniasis

**DOI:** 10.4103/0974-777X.62876

**Published:** 2010

**Authors:** Susanne Nylén, Shalini Gautam

**Affiliations:** *Department of Microbiology Tumor and Cell biology, Karolinska Institutet, Stockholm, Sweden*; 1 *Department of Medical Sciences, Benares Hindu University, Varanasi, India*

**Keywords:** *Leishmania*, Immunology, T cells, Innate immunity, Human, Apoptosis, Vaccine

## Abstract

*Leishmania* parasites have been widely used in experimental models to understand generation, maintenance and failure of immune responses underlying resistance and susceptibility to infection. The clinical outcomes of *Leishmania* infection depend on the infecting species and the immune status of the host. Noticeably most people exposed *Leishmania* never develop overt disease. Understanding the immunological events that result in failure or successful control of the parasites is fundamental to both design and evaluation of vaccines and therapies against the leishmaniases. Recent studies visualizing immune response to *Leishmania major* in the skin have given new insights into the different immune cells acting as hosts the parasite during different stage of infection. Control of *Leishmania* infection and disease progression has been associated with generation of T-helper (Th) 1 and Th2 responses respectively. Though still valid in several aspects, the Th1/Th2 paradigm is an oversimplification in need of revision. Th2 polarization has never explained severity of human leishmanial disease and a number of other T-cell subsets, including regulatory T- and Th17- cells, have important roles in susceptibility and resistance of both experimental and human leishmanial disease. This review gives an updated overview of immunological response considered to be of importance in protection, susceptibility, disease progression and cure of leishmaniasis, with a special emphasis on human diseases.

## INTRODUCTION

All natural *Leishmania* infections start when *Leishmania* promastigotes are injected into the skin dermis of humans and other warm-blooded animals. To survive, the parasite must resist exposure to host serum component and destruction by innate immune cells present in or rapidly recruited to the skin. The skin is a complex immunological organ in which multiple innate immune cells [Table 1] function to protect the host from infectious pathogens. Normal skin of adult humans also contains a substantial number of T cells, nearly twice that present in the circulation,[1] which may play an important role in the local response.

**Table 1:** Innate immune cells in the skin and their role in leishmanial disease (Leish)

Cell type	General function	Observations in murine Leish	Observations in Leish *in vitro*
**Keratinocytes**	Sensors of injury & infection	-	Source of IL-10, associated with PKDL[111]
*Location: epidermis*			
**Langerhans cells**	Antigen presentation in certain infections	Uncertain, not necessary for induction of Th1 responses[33]	Correlation between high LC density and acute cutaneous *L. tropica* disease[112]
*Location: epidermis*	Induction of peripheral tolerance		
	Th2 induction		
	Cross priming of naïve CD8+ T cells		
**Dermal DC**	Immune surveillance	Sensors of infection[33]	-
*Location: dermis*	Antigen presentation		
	Cross presentation to CD8+ T cells		
**Dermal macrophages**	Antimicrobial activity and production of pro- and anti-inflammatory mediators	Can act as host cells [8,32]	Host cells (non-human primate skin)[6]
*Location: dermis*			
**Plasmacytoid DC**	IFNα production	Leishmania loaded pDC can induce protective immunity[113]	-
*Location: dermis*	Activation of NK cells, B cells T cells and myeloid DC cells		
**Mast cells**	Regulating later Inflammatory response by Neutrophils	Sentinels, contribute to DC recruitment[114] Tissue pathology[115]	Elevated numbers in MCL lesion[117]
*Location:Dermis*		Susceptibility[116]	Possibly an association with wound healing[118]
**Monocyte derived**	Inflammatory cells	Induction of protective immunity[7]	Species dependent production of IL-12, co-stimulation[36]
**Inflammatory DC**	T cell stimulation Production of IL-12, iNOS and TNFα	Primary cells harboring parasites in later stages of disease development (healing mice)[28]
*Location: Inflamed dermis*			
**Neutrophils polymorph nucleated cells (PMN)**	Uptake and destruction of pathogens	Temporary early major host cells facilitating L. major infection[8]	*Human PMN can kill promasitigotes and amastigotes.[14]
			Silent Transfer of parasites into macrophages[13]
*Location: dermis*		Protective[16]	Found in lesions[18,20]
		Tissue pathology – in later stages of disease[22,23]	Can harbor parasites in VL[9]
**NK cells**	Early source of IFNγ	Contribute to early resistance against the parasite[37,119]	Associated with protection and cure[43,45]
*Location: Inflamed dermis*			

The general functions of cells have been adapted from Nestle *et al*.[110]

The interaction with the complement system depends on the developmental stage and the species of the parasite. In short, exponentially multiplying log-phase promastigotes are sensitive to complement mediated lysis and perform poorly in experimental infections while metacyclic promastigotes and amastigotes are more resistant and more infective.[2,3] However, no parasite is completely resistant to physiological plasma complement levels.[4] Prompt infection of susceptible host cell may be essential for survival. While surface deposition of complement can cause destruction, *Leishmania* can use deposited C3b, which is rapidly converted into iC3b, to facilitate parasite entry into macrophages and neutrophils via complement receptor (CR)3.[3,5]

## NEUTROPHILS – TROJAN HORSES, EXPLOITED INTERMEDIATE HOST CELLS OR PARASITE KILLERS?

*Leishmania* infection has been assumed to be initiated by direct parasitization of skin resident macrophages[6] whereas uptake by skin DCs has been linked to priming and shaping the T cell response.[7] Recent studies have, however, slightly changed this view; *in vivo* imaging of sandfly transmitted *L. major* infection revealed neutrophils as the first cells to be infected by the parasites.[8]

Neutrophils are primary antimicrobial effector cells, with the main function to phagocytose and destroy invading pathogens. Neutrophils are rapidly recruited to sites of the body where tissue damaged has occurred, such as the site of a sandfly bite. Most microorganisms are rapidly killed when taken up by neutrophils, but a few mostly intracellular pathogens, can survive the destructive milieu of these cells.

Following sandfly transmission or needle inoculation with *L. major,* invading neutrophils were observed to rapidly and efficiently capture parasites.[8] Infiltrating neutrophils did not destroy the parasites, instead they facilitated infection as depletion of neutrophils prior to infection reduced the parasites load and delayed onset of disease.[8] Infection of neutrophils is transient and within a week post infection macrophages/monocytes take over as the primary host cell.[8] However, in human VL neutrophils have been reported to harbor parasites during active disease.[9,10] It may be noted that human blood is much more neutrophil rich than that of mice.[11]

Laskay and colleagues have supported by *in vitro* studies showing that *Leishmania* infected apoptotic neutrophils can be taken up by macrophages that allow parasites to thrive, proposed a model in which neutrophils, act as “Trojan horses” for *Leishmania*.[12,13] Apoptotic neutrophils are normally cleared without triggering activation of macrophages. Thus, uptake of infected apoptotic neutrophils could facilitate silent entry and infection of macrophages. *Leishmania* may also extend the life span of a neutrophil by delaying apoptosis, suggested to give monocytes time to infiltrate the site of infection and become infected by apoptotic neutrophils.[13] However, the *in vivo* images by Peters *et al.* were not able to capture neutrophils uptake by monocytes/macrophages.[8] Instead parasites were observed to egress dying neutrophils, to invade macrophages. Regardless if neutrophils act as Trojan horses or not, compelling evidence indicates that the *Leishmania* parasite, in the early infection phase, can both evade and exploit neutrophils to ensure its survival.

Nevertheless, when appropriately activated, neutrophils can kill intracellular pathogens such as *Leishmania*[14] and there are several reports suggesting that neutrophils play a role in early protection against Leishmanial disease.[15–17] *Leishmania amazonensis* promastigotes can induce and be killed by neutrophils extracellular traps (NETs). The same would appear to be the case for amastigotes, albeit not to the same extent as promastigotes. More interestingly meshes of DNA and elastate suggestive of NET were found in skin biopsies of patients.[18] Furthermore, in a mouse model of *Leishmania donovani* infection, using amastigotes for infection, neutrophils were found to have a protective function.[16]

The capacity of neutrophils to function as immune evasion targets probably depend on the genetic background of the host, the parasite strain and the developmental stage of the parasite used.[19] While metacyclic promastigotes may survive in neutrophils, non-metacyclic ones can rapidly be killed. Neutrophils may act in disease-stage specific way, being permissive hosts for metacyclic promastigotes while contributing to the over-all inflammatory and parasitocidal response in active lesions. In both human and murine leishmaniasis neutrophils are prominent infiltrates in lesions;[20,21] their presence at the site of infection can cause immune mediated tissue pathology.[22,23]

## MANIPULATING ANTIGEN PRESENTING CELLS A KEY STRATEGY FOR SURVIVAL

For a productive infection, the *Leishmania* parasites need to establish in macrophages. Macrophages possess potent antimicrobial functions and activated macrophages can kill *Leishmania.* To survive the parasite need to avoid macrophage activation and recognition by T cells. The ability to survive in macrophages is partly stage specific with metacyclic promastigotes having better capacity to survive compare to pro-cyclic promastigotes.[3] The parasites have several strategies by which macrophage activation can be prevented including: silent entry utilizing engagement of non-triggering receptors such as CR1 and the phosphatidyl serine (PS) receptor;[24,25] more direct inhibition of macrophage function by interfering with NFB transcription and IL-12 production, down regulation of MHC class II; promoting production of regulatory cytokines like IL-10 and TGFβ.[26,27]

Dermal macrophages are readily infected with *Leishmania* and permit differentiation and growth and may initiate the infection.[6] However, most targets for *Leishmania* would appear to be infiltrating monocytes/macrophages, which enter the site of infection one to two days post infection.[8,28] Interestingly, recent studies show that macrophages may not be the main host cells for the parasites in chronic stages of healing disease. In chronic self-healing *L. major* infection (in C57Bl/6) mice) TNF and iNOS producing CD11b+CD11c+Ly6C+MHC-II+DC (TIP-DC), which most likely are derived from monocytes, host the majority parasites in the skin.[28,29] If this is related to cure and generation of a protective Th1 response and/or preparing the parasite for transmission to blood feeding sandflies, is not known.

## APOPTOSIS – A WAY TO AVOID IMMUNE ACTIVATION AND PROMOTE SURVIVAL

**Figure 1:**
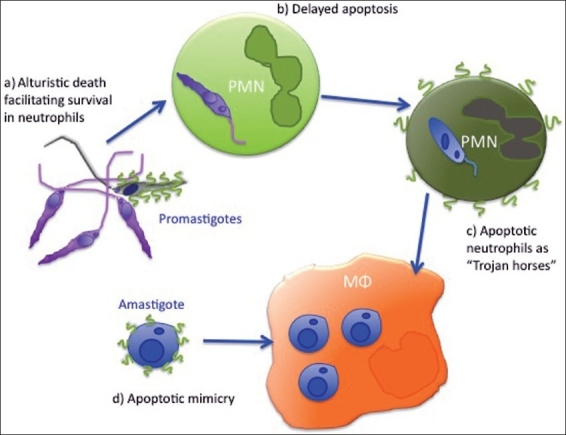
Apoptosis - a way to avoid immune activation and promote survival; a) Sandflies inject apoptotic parasites together with viable. Apoptotic promastigotes facilitate infection and prevent activation of neutrophils (PMN).[108,109]; b) Parasites delay neutrophil apoptosis, giving monocytes/macrophages (Mφ) time to enter the site of infection; c) Silent entry of parasite into macrophages via apoptotic neutrophils.[12]; d) Viable amastigotes expose PS and mimic apoptotic cells. This facilitates internalization and increases macrophage susceptibility to leishmanial growth[25]

Exposure of PS is fundamental for the non-inflammatory phagocytosis of apoptotic cells. Recognition of this phospholipid by macrophages induces TGFβ secretion, IL-10 synthesis and inhibits NO production. Utilizing apoptotic cells as vectors or mimicking mammalian apoptotic cells is a strategy to escape host protective inflammatory response. *Leishmania* parasites have evolved several strategies to use apoptosis to it advantage [Figure 1].

## DENDRITIC CELLS ORCHESTRATING THE IMMUNE RESPONSE

*Leishmania* parasites-DC, interactions are complex, inconsistent and may lead to the control of infection or progression of disease. Activation of DC varies in quantity and quality depending upon developmental stage and the species/strain of *Leishmania* as well as DC cell subset and exogenous stimuli involved in different studies.[30]

Several different types of DC with different location and probably different function exist in the skin. The first studies of murine skin DC indicated epidermal Langerhans cells (LC) as the important cells for sensing, uptake and transport of *Leishmania* to the lymph node.[31] More recent studies have shown that it is dermal DCs that are involved in the early recognition of the parasite. Dermal DC can efficiently take up and incorporate parasites in vacuoles[32] and have been suggested by some to act as principal antigen presenting cells in leishmaniasis,[33] while other suggest lymph node resident DC as the initiators of the immune response.[34]

*Leishmania* have evolved several strategies to avoid or dampen DC, while some appear general to *Leishmania* other tend to be species related.[30] This may be, in part, the explanation for the severity of disease caused by the different species. In general, more papers report inhibitory effects of *L. donovani* and the South American species (*L. amazonensis*, *Leishmania braziliensis*) compared to *L. major*. In human cells *L. donovani* has been suggested to block maturation of human DC.[35] Production of IL-12 by DC, which is essential for the initiation of a protective immune response in mice (and probably also in humans) is differently affected by *L. donovani* and *L. major*: while uptake of *L. major* by human monocyte derived DC efficiently prime DC for IL-12 production uptake of *L. donovani* does not.[36]

## A RECOVERED ROLE FOR NATURAL KILLER (NK) CELLS IN INDUCTION OF IMMUNITY

Together with phagocytes, NK cells represent the first line of defense against pathogens by two principal mechanisms, cytolytic destruction of infected cells and secretion of pro-inflammatory cytokines (e.g. IFNγ, TNFα).

Early studies of experimental leishmaniasis in C3H/HeN mice indicated that IFNγ production by NK cells was important for generation of protective immune responses and control of infection. Subsequent studies, which of note, were mainly done in mice on C57Bl/6 background, demonstrated, however, that NK cells are not required for generation of adequate T helper type-1 response and protective immunity. Their presence however, may delay onset of disease as Balb/c mice lacking NK cells develop lesions faster and harbor more parasite.[37] Thus, NK cells can serve a function in control of *Leishmania* burden during early phases of infection through their ability to rapidly respond with IFNγ production. *In vitro*, human NK cells have been shown to have the ability to be directly activated to IFNγ production by *Leishmania* promastigotes or their LPG.[38,39]

A new interest in NK cells and *Leishmania* infection has evolved from the interaction between these cells and DC. Activated NK cells promote DC maturation, while they may kill autologous immature DC.[40] DC can on the other hand efficiently prime resting NK cells. *In vitro* resting NK cells have been shown to promote activation of DCs pre-infected with *L. amazonensis* promastigotes and these activated DCs can, in turn, mostly via cell contact-dependent mechanisms stimulate NK cells.[41] *L. major* infection induces NK cells to secretion of IFNγ and *in vivo* imaging has shown that NK cells are recruited to the paracortex, a strategic area in the lymph node, where they can interact with DC and regulate co-localized CD4 T cells responses.[42]

In patients, NK cell number and activity has mainly been associated with protection against or healing of disease. Patients with active leishmaniasis (cutaneous and visceral) have been reported to have a reduction in the frequency of peripheral NK cells[43,44] and recently an increased frequency NK cells, following immunotherapy, in a *L. amazonensis* diffuse cutaneous leishmaniasis (DCL) patients was associated with cure.[45]

## EFFECTOR T CELLS IN CONTROL AND FAILURE OF LEISHMANIAL INFECTION

Protective immune responses against *Leishmania,* in self-healing strains of mice (C57BL/6, C3H, CBA), are intimately linked to development of a Th1 response and IFNγ production. In experimental self-healing disease DC are stimulated to produce IL-12, which drives the generation of Th1 cells (effector and memory). T cell dependent IFNγ in turn activates macrophages to upregulation of iNOS and NO production, which results in killing of intracellular parasites and control of disease.[46,47] Disease progression has to a large extent been viewed as development of Th2 responses and IL-4 based on studies of *L. major* infection in Balb/c mice. The polarized Th1/Th2 responses in mice have been more thoroughly reviewed by others[48] and is not the scope of this review.

Most data point to the fact that same or similar Th1 dependent mechanisms are involved in control of human disease. Self-healing forms of leishmaniasis and cure of VL is typically accompanied by parasite specific proliferation and IFNγ production. Human macrophages are activated to kill intracellular parasites by IFNγ and exogenous IFNγ can promote cure of human CL.[49] Though Th2 responses can act in favor of the parasite, polarized Th2 response has never been able to explain non-curative or visceralizing human disease. Th2 independent disease progression is also supported by studies on non-healing disease in the Th1 phenotypic B6 mice.[50] In this context it can also be noted that in patients with VL the effect of IFNγ administration was limited[51] and in human CL, IFNγ production by CD4+ cells, alone, in response to *Leishmania* antigens is not predictive of protection or disease development.[52] This indicates that other mechanisms acting in synergy with IFNγ or counteracting the effects of IFNγ are as important.

## ADVANCING THE TH1/TH2 PARADIGM: TH17 AND REGULATORY T CELLS AND THEIR ROLE IN PATHOLOGY OF LEISHMANIAL DISEASES

Th17 and Treg are today widely accepted subsets with important functions in induction and control of the inflammatory response. Both Th17 and Treg have a greater degree of plasticity in their differentiation decision, as compared to conventional Th1 and Th2 cells, enabling response to signals provided by the environment in which they reside.[53]

Th17 cells are pro-inflammatory T helper cells, hallmarked by their ability to secrete IL-17. IL-17 is involved in recruitment, migration and activation of neutrophils and Th17 cells have an important function in protecting surfaces against certain extracellular bacteria and fungal pathogens, but can also mediate severe immune pathologies.[54]

In experimental leishmaniasis Th17 cells have been associated with tissue destruction: IL-17 deficient Balb/c mice develop smaller lesions, have decreased CXCL2 accumulation and fewer neutrophils in lesions as compared to wild type, while elevated IL-17 conferred no reduction in parasite load.[22]

Interestingly, a recent study of human VL linked IL-17 and IL-22 (a pro-inflammatory cytokine, produced by Th17 and NK cells), to protection against human *kala-azar* caused by *L. donovani. L. donovani* was furthermore shown to stimulate generation of cells producing IL-17, IL-22 as well as IFNγ by human T cells.[55] IL-27 is an IL-12 related cytokine, produced mainly by macrophages and DC, thought to be important in regulation of Th17 cells. In C57Bl/6 mice IL-27 is important in early Th1 development, mediating suppression of the early IL-4 burst that occur in B6 mice.[56] IL-27 also has anti-inflammatory properties, mediated through the ability of IL-27 to suppress Th17 cells[57] and induction of naïve human CD4 cells to IL-10 production.[58]

Interleukin-27 receptor deficient mice display enhanced resistance to *L. donovani* infection[59] and findings of elevated levels of IL-27 together with low RoRγT/IL-17 in VL patients before treatment, implicate IL-27 in VL pathogenesis.[60] However mice deficient in IL-27R develop severe liver immunopathology when infected with *L. donovani*[59] and more severe cutaneous lesions in infection with non-healing *L. major*.[57] Both studies showed that CD4 T cells were linked to pathology, in the latter study IL-27 was found to regulate both IL-10 and IL-17, and tissue pathology was associated with IL-17 producing T cells. Thus, the elevated levels of IL-27 in human VL may serve an important function suppression of IL-17 producing CD4 T cells and subsequent tissue damage by neutrophils.

## REGULATORY T CELLS AND PARASITE PERSISTENCE

Regulatory CD4 T cells can broadly be divided into two categories - natural Foxp3+ (CD4+CD25^high^) regulatory T cells (nTreg) that arise in the thymus and inducible regulatory T cells generated in the periphery, the latter can be both, Foxp3+ (iTreg) and adaptive FoxP3-, type-1 regulatory T cells (Tr1). All regulatory T cells act to counteract inflammatory immune response to limit tissue damage and absence of regulatory T cells is linked to a number of autoimmune conditions.

The skin (and other epithelial surfaces) has a high frequency of steady state, nTreg that function to suppress the generation of harmful immune response to infectious and non-infectious antigens to, which the skin is regularly exposed. The presence of these cells may, however, precondition the skin for survival of *Leishmania* parasites and favor long-term parasite survival.[61] In humans CD4+CD25+ Treg cells are found in cutaneous lesions[62] and elevated intra lesional FoxP3 and IL-10 have been associated with unresponsiveness to treatment during *L. amazonensis* infection.[63]

IL-10 is a cytokine intimately linked with disease progression of both murine and human *Leishmania* infection.[64] Experimental models have clearly demonstrated the central role played by IL-10 in pathology and parasite persistence.[65–67] In human VL, elevated levels of IL-10/IL-10 mRNA are found systemically as well as in spleen, bone marrow and lymph nodes. A role for IL-10 in human VL pathology is supported by studies indicating that IL-10 blockade can enhance VL PBMC IFNγ responses and inhibit VL serum promoted parasite replication in macrophages.[44,68,69] However, if the IL-10, as assumed, is a major suppressor of effector T cell in VL patients, remains to be proved. In human CL, elevated IL-10 has been demonstrated in lesions.[70–72] A recent genetic analysis of IL10-819C/T polymorphism, in the IL10 promoter, showed that the C allele, which is linked to higher levels of IL-10 production, is associated with increased risk of developing cutaneous lesions in populations exposed to *L. braziliensis.*[72]

All regulatory T cells can be sources of IL-10. In leishmanial infection most data point to antigen-induced Foxp3- T cells, producing IL-10, as being responsible for delayed healing associated with disease progression.[50] In line with this T cells other than those expressing FoxP3 would appear to be the main source of IL-10 in human VL.[43,44] The B6 mouse model of non-healing disease indicates that Th1, which secrete IFNγ cells, can be the main source IL-10.[50] IL-10 secretion by Th1 cells is a self-regulating mechanism evolved to minimize T cell mediated immune-pathology.

Central cytokines in healing and progression of leishmanial disease are summarized in Table 2.

**Table 2:** Expanding the Th1/ Th2 paradigm: A brief summary of central cytokines in healing and progression of Leishmania infection

Cytokine	Producer/s	Function in murine Leish	Human Leish (correlation)
IL-4	**Th2 cells**	Inhibition of Th1 responses	Some association with non-healing CL and VL[120,121]
	Mast cells basophils	Responsible for progression in Balb/c mice[48]	
IL-10	Many, including **monocytes/macrophages, T cells** and epithelial cells	Promote parasite persistence	Associated with visceral and non-healing disease.[64,111,122]
		Down regulation of macrophage function. Counter act Th1 cells[65,66,99]	
IL-12	**Dendritic cells**	Required for induction of protective Th1 response[123]	Addition to VL PBMC induce IFNγ and cytotoxic Monocytes response[124,125]
	Neutrophils		
	B cells		
IL-17	**Th17 cells,** neutrophils	**Disease progression in susceptible Balb/C mice**[22]	Associated with protection from disease[55]
IL-22	**Th17 cells**	-	Associated with protection from disease[55]
	NK-22 cells		
IL-27	**Dendritic cells** Monocytes	Fewer parasites, but more tissue pathology due to impaired regulatory response[57,59]	Associated with active VL[60]
	Macrophages		
IFNγ	Many, most importantly **Th1** cells and NK cells	Required for protective responses, KO mice cannot control infection[48]	Antigen specific INFγ response by PBMC are associated with cure and protection[126]
			May promote cure of CL[49]
TNFα	Many, mainly **macrophages**	Required for control of most, leishmania strains.[127]	Associated with protection and cure[126]
		Cause tissue destruction and loss of splenic architecture in experimental VL[128]	Case reports of VL in TNF antagonist treated patients[129]
			High levels associated with tissue pathology[130]
TGFβ	**Monocytes /macrophages**	Regulatory function associated with disease progression.[131]	Associated with non-healing phenotype (MCL, PKDL)[131,134]
	T (reg) cells Chondrocytes	Suppression of IFNγ by NK cells[132]	
		Act in synergy with IL-10[133]	

Important producers of the respective cytokines in leishmanial disease

## CD8 T CELLS ARE PROTECTIVE AND DESTRUCTIVE

The role of CD8+ T cells is still not completely defined in *Leishmania* infection. Though a number of early reports suggested a role for CD8+ cells in immunity against *L. major* infection,[73,74] CD8+ T cells, were for a long time thought to play a secondary role as CD8 cells alone could not induce protective immunity and CD8 defective mice, were able to control infection.[75] However, Belkaid *et al*. later demonstrated that CD8 cells actually were required for healing when C57BL/6 mice were infected with a low, and more physiological relevant, dose of parasites and in experimental infection with *L. donovani* both CD8 and CD4 can on their own cells prevent reactivation of disease.[76,77]

CD8+ T cells participate in protection against pathogens by two major mechanisms: production of cytokines (IFNγ and TNF-α) and by direct killing of infected cells. In *Leishmania* infection the main contribution of CD8 T cells in immunity is considered to be through IFNγ production. Cytotoxic T-lymphocyte (CTL)-mediated mechanisms in the regulation and control of *Leishmania* infection remain largely unexplored.[78] Perforin (together with IFNγ) has, however, been suggested as an important effector molecule in vaccination induced immunity against *L. amazonensis*.[79] Both murine and human *Leishmania* infection can prime CD8 T cells for killing of antigen pulsed macrophages.[80,81] *In vivo* studies have moreover indicated that the Fas-Fas ligand (L) pathway contributes to healing of lesions induced by *L. major*,[82] as Fas- and FasL-deficient mice cannot control infection despite upregulation of IL-12 and NO production. Moreover, CD95 is required for the early control of parasite burden in the liver of *L. donovani* -infected mice.[83] In contrast, elevated levels of Fas in human CL lesions have been suggested to contribute to ulcer formation.[84]

CD8+ T-cells have been associated with both cure and pathology in human leishmaniasis: An expanded CD8+ cell population was observed in the draining lymph node prior to ulcer development, implicating CD8 mediated immunity in the early containment of *Leishmania* infection.[85] An increase in responding CD8+ cells has been associated with cure of *L. braziliensis* CL.[86,87] Exacerbated CD8+ activity, in addition to a poor regulatory response, could however, underlie an unfavorable fate with regard to MCL. Recruitment of CD8+ T cells expressing granzyme associated with lesion progression of CL caused by *L. braziliensis* and more CD8 cells were found in relapse cases.[88,89] Accumulation of CD8 cells have also been linked to PKDL.[90]

CD8 T cells can just like CD4 T cells have natural or acquired regulatory properties.[91] IL-10 producing CD8 cells of memory phenotype have been identified in humans infected with *Leishmania guanyensis*.[92] The function of these IL-10 producing CD8 T cells in leishmanial disease is unknown.

## B CELLS AND ANTIBODY RESPONSES: HARMFUL OR PROTECTIVE

B cells and antibodies are generally not considered to be of major importance in protective immunity against *Leishmania*. Antibodies are not effective at killing the parasite as it hides inside the parasitophorus vacuole and antibody responses in self-healing cutaneous disease are very modest.

High levels of *Leishmania* specific antibodies are observed in patients with VL and other severe forms of leishmanial disease and there are accumulating evidence that B cells and antibodies correlate with pathology.

A model where immunoglobulin (IgG) promotes infection by inducing IL-10 was proposed by Kane and Mosser, who showed that IgG coated amastigotes (*L. major*) could ligate Fc-receptors on murine macrophages and induce IL-10 production.[67] In support of this models *in vivo* studies found that Fc-deficient mice infected with *L. amazonensis* produce less IL-10 and are less susceptible to infection.[93] Moreover, a regulatory role for B cells has been suggested in a VL model demonstrating that B cell depleted animal exhibit extensive neutrophil mediated pathology.[23]

There is still much to learn about how antibodies function in leishmaniasis and it should not be ruled out that certain antibodies might contribute to protection. Immunization with, the for dogs licensed vaccine, Leishmune, which confers some protection against leishmaniasis, result in seroconversion and an increase in the proportion of B cells.[94]

## IMMUNE RESPONSES ASSOCIATED WITH VISCERAL DISEASE

Infection with *L. donovani* and *Leishmania infantum* results in the establishment of the parasite in the liver, spleen and bone marrow in mice. The liver is, in most mouse strains, the site of an acute resolving infection associated with the development of inflammatory granulomas around infected Kupffer cells, and resistance to re-infection. In contrast to the liver, parasites persist in the spleen. Persistent parasites are characterized by lack of granuloma formation, splenomegaly, enhanced hematopoietic activity and disruption of lymphoid tissue micro-architecture, the latter postulated to contribute to the immuno-compromised status of the host. Splenic pathology is linked to high levels of both TNFα and IL-10. TNFα mediates destruction of marginal zone macrophages and gp38+stromal cells, while IL-10 is responsible for impaired DC migration into T cell areas and defective T cell priming. Furthermore, the altered stromal cells function can promote development of IL-10 producing DC, with immuno-regulatory properties (reviewed in[95]).

The mechanisms underlying the failure to control growth and spread of parasites in human VL are less well understood. Most humans infected with visceralizing *Leishmania* species never develop disease. How control is mediated in these people is largely unknown. Absence of antigen specific Th1 responses, in peripheral blood mononuclear cells of VL patients is, thought to be, causally related to disease progression. However, there appears to be no inherent defect in antigen induced Th1 response as patient cell respond after cure and the finding that patients express elevated levels of IFNγ mRNA in T cells, in lesional tissue and the multiple pro-inflammatory cytokines, found systemically and at the site of infection indicates that neither can their immunological defect simply be explained by immune tolerance or Th2 polarization. Most clinical evidence point to that the host regulatory response, generated to limit collateral tissue damage, promotes spread and growth of microorganism in chronic infections, like VL. Human VL is clearly associated with elevated levels of the regulatory cytokines IL-10.[64] The inhibitory effect of IL-10 on macrophage may be detrimental, as IL-10 renders macrophages unresponsive to activating cytokines. In addition IL-10 may contribute to loss of important effector T cells in VL. It is still unclear if patients with VL have developed antigen specific T cell responses that are suppressed by regulatory responses or if antigen specific T cell responses are never appropriately generated.

## THE QUEST FOR A VACCINE AGAINST LEISHMANIASIS

After clinical cure from leishmanial disease people are considered to have acquired lifelong immunity to infection with the same parasite, making vaccination a feasible measure. However, developing a human vaccine against *Leishmania* has proven difficult despite the many vaccine candidates reported to be protective against murine leishmaniasis. In humans, vaccination with autoclaved *L. major* (considered protective in mice) adjuvanted with BCG has had limited success.[96] The only reliable preventive measure against clinical disease remains leishmanzation (LZ), an ancient method where viable parasites are inoculated at a covered part of the body to protect against subsequent disfiguring disease. While this method may be used to protect against mild cutaneous disease, it cannot be used against VL. Moreover, LZ has been questioned due to fear of non-healing and disseminating ulcers.

Vaccination in mice may, however, not be as straight forward as initially thought. It was recently shown that inoculation of killed *Leishmania* in mice that resolved their primary *L. major* infection resulted in rapid and relatively sustained loss of infection-induced immunity.[97] Moreover, while vaccination with killed parasites plus CpG adjuvant confer protection against needle challenge, vector transmitted infection can abrogate this protective immunity. Only live infection was able to protect against subsequent vector mediated transmission.[98] Indeed, studies indicate that parasite persistence may be required for maintenance of protective T cell immunity and “sterile” cure would again make the animal permissive for disease.[61,99]

Vaccination with attenuated parasites may be the solution for preventing leishmanial disease. Several methods have been used to develop live attenuated *Leishmania* parasites including long term *in vitro* cultures, selection for temperature sensitivity, chemical mutagenesis and irradiation.[100] Attenuated lines have been shown to confer substantial protection in animal models, but undefined mutations and concerns regarding conversion back to virulence make them unsuitable for human use. Targeted elimination of virulence or essential genes could, if carefully done, solve this problem. Recently, a *L. donovani* strain completely deficient in the centrin gene, (required for growth in the amastigote stage) was found to be both safe and protective against both homologous and heterologous parasites in rodent models.[101] While more studies are required this may be an attractive vaccine candidate against VL.

## COINFECTION – A REALITY RARELY CONSIDERED IN EXPERIMENTAL MODELS

The poor populations, which are mainly affected by leishmaniasis, are also plagued with many other chronic infections such as helminths, other protozoa, tuberculosis and HIV/AIDS.

The visceralizing species of *Leishmania* can in many aspects be considered as opportunistic infections, and patients with HIV are at much greater risk of developing VL.[102] From an immunological perspective the two agents ability to escape and manipulate the immune response seem to a large extent work in synergy, resulting in a dangerous liaison where the immune system rapidly can be exhausted and control of pathogens lost.[103–105] HIV/AIDS can also slow down diagnosis of VL as antibody based tests may not be indicative of disease in AIDS patients. Interestingly, while HIV may have a profound impact on VL the evolution of purely cutaneous disease would only seem to be only moderately affected and *L. major* have not been reported to visceralize in HIV patients.[102]

Helminths are another group of pathogens, which may favor survival of *Leishmania* parasites by the Th2 and regulatory immune responses they induce. In this respect, it has been shown that mice have better capacity to deal with *L. donovani* and *L. major* infection in the absence of *Schistosoma mansoni* infection.[106,107]

Coinfection are a reality that need to be considered when developing and evaluating vaccines against human leishmaniais in endemic populations. Treating co-infections, in particular worms may be a measure to enhance vaccine and therapeutic efficacy.
